# Causative species of nontuberculous mycobacterial lung disease and comparative investigation on clinical features of *Mycobacterium abscessus* complex disease: A retrospective analysis for two major hospitals in a subtropical region of Japan

**DOI:** 10.1371/journal.pone.0186826

**Published:** 2017-10-23

**Authors:** Hiroaki Nagano, Takeshi Kinjo, Yuichiro Nei, Shin Yamashiro, Jiro Fujita, Tomoo Kishaba

**Affiliations:** 1 Department of Respiratory Medicine, Okinawa Chubu Hospital, Okinawa, Japan; 2 Department of Infectious Diseases, Respiratory, and Digestive Medicine, Graduate School of Medicine, University of the Ryukyus, Okinawa, Japan; National Institute of Infectious Diseases, JAPAN

## Abstract

Nontuberculous mycobacteria (NTM) lung disease is increasing globally. Although the etiological epidemiology of NTM is different across regions, *Mycobacterium avium* complex (MAC) is the leading cause of NTM lung disease in most countries, including mainland Japan. Okinawa is located in the southernmost region of Japan and is the only prefecture categorized as a subtropical region in Japan, it is therefore likely the etiological epidemiology of NTM lung disease is different from mainland Japan. From 2009 to 2015, the medical records of patients, with respiratory specimens positive for NTMs, visiting or admitted to two Okinawan hospitals, were retrospectively analyzed. NTM lung disease cases were defined according to the American Thoracic Society criteria and patient epidemiology and clinical information were evaluated. Results indicate four hundred sixteen patients had bacterial cultures positive for NTM. The most common NTM was *M*. *abscessus* complex (MABC) (n = 127; 30.5%), followed by *M*. *intracellulare* (n = 85; 20.4%). NTM lung disease was diagnosed in 114 patients. Of these cases, MABC was most common (n = 41; 36.0%), followed by *M*. *intracellulare* (n = 31; 27.2%). Chronic obstructive pulmonary disease (COPD) and tracheostomy patients were more likely to develop MABC than MAC lung disease. Multivariate analysis showed a probable association between COPD and MABC lung disease. Chest computed tomography (CT) evaluation revealed bronchiectasis, nodules, and consolidation were less frequently observed in MABC patients compared with MAC patients. Our data suggests Okinawa may be one of the few places where MABC is the predominant pathogen causing NTM lung disease and our results add new insight to MABC lung disease, which is not yet well understood.

## Introduction

Nontuberculous mycobacteria (NTM) are ubiquitous bacteria widely distributed in the environment. NTM can cause a variety of infectious diseases in humans and NTM-induced lung infection is increasing globally [1, 2]. A recently published article shows that the incidence of pulmonary NTM disease in Japan has increased dramatically and cases of pulmonary *M*. *avium* complex (MAC), *M*. *kansasii*, and *M*. *abscessus* complex (MABC) disease were 13.1, 0.6, and 0.5 cases per 100,000 person-years, respectively [3]. The NTM species isolated from patients with NTM lung disease are geographically diverse, nevertheless MAC is the most common species in most countries [4].

Although rapidly growing mycobacteria (RGM), represented by MABC, *M*. *fortuitum*, *and M*. *chelonae*, are not common pathogens of NTM lung disease, MABC was frequently isolated from NTM lung disease patients in South Korea and Taiwan [3–6]. A single-center study reported from Taiwan, located 500 km south of Okinawa and also categorized as a subtropical region, showed that MABC was the most common species (44.8%) followed by *M*. *fortuitum* (23.9%) isolated from NTM lung disease patients; thus, RGM accounted for approximately 70% of the caseload [6]. Because of the subtropical climate of Okinawa is different than that of mainland Japan and is more similar to Taiwan, we hypothesized that the isolation rate for certain causative species of NTM lung disease in Okinawa and mainland Japan is also different. In this study, we retrospectively investigated the causative species of NTM lung disease and the clinical features of MABC induced lung disease, in two representative hospitals from Okinawa.

## Materials and methods

### Data collection

Between January 2009 and December 2015, patients, with NTMs cultured from respiratory specimens, visiting the outpatient department or admitted to the ward in the Okinawa Chubu Hospital (550 hospital beds) or the University of the Ryukyu Hospital (600 hospital beds), had their medical records retrospectively reviewed. NTM lung disease cases, defined according to the American Thoracic Society (ATS) criteria [7], were identified, underwent etiological analysis by species, and had other clinical information extracted. The clinical background, symptoms, and chest computed tomography (CT) findings of MABC and MAC infected patients were also compared.

### Identification of NTM

Respiratory specimens were cultured with 2% Ogawa agar and the resulting bacterial colonies were collected for species identification. The resulting suspension liquid was tested using a DNA-DNA hybridization method from a commercially available identification kit (KYOKUTO Pharmaceutical Industrial Co. Ltd., Tokyo, Japan).

### Evaluation of chest CT

Chest CT was evaluated by two experienced chest physicians. Abnormal findings were categorized into; (1) bronchiectasis, (2) nodules, (3) consolidation, (4) cavity, (5) ground-glass opacity, and (6) linear scarring, according to previous reports [8, 9]. Patients could be categorized with more than one finding.

### Statistical analysis

Differences among patients’ background, symptoms, and chest CT images were compared for MABC and MAC patients by Fisher’s exact test. The difference in age between these two groups was analyzed by Wilcoxon/Kruskal-Wallis test. A two-sided *p*-value of <0.05 was considered to be statistically significant. Previously reported risk factors [10–12] for the development of NTM lung disease and possible risk factors revealed by this study were evaluated using logistic regression. All data were analyzed with JMP ver.12 (SAS Institute Inc., North Carolina, USA).

### Ethics

The Institutional Ethics Committees of the Okinawa Chubu Hospital and the University of the Ryukyus approved this study.

## Results

### Pathogen distribution

**Fig 1:**
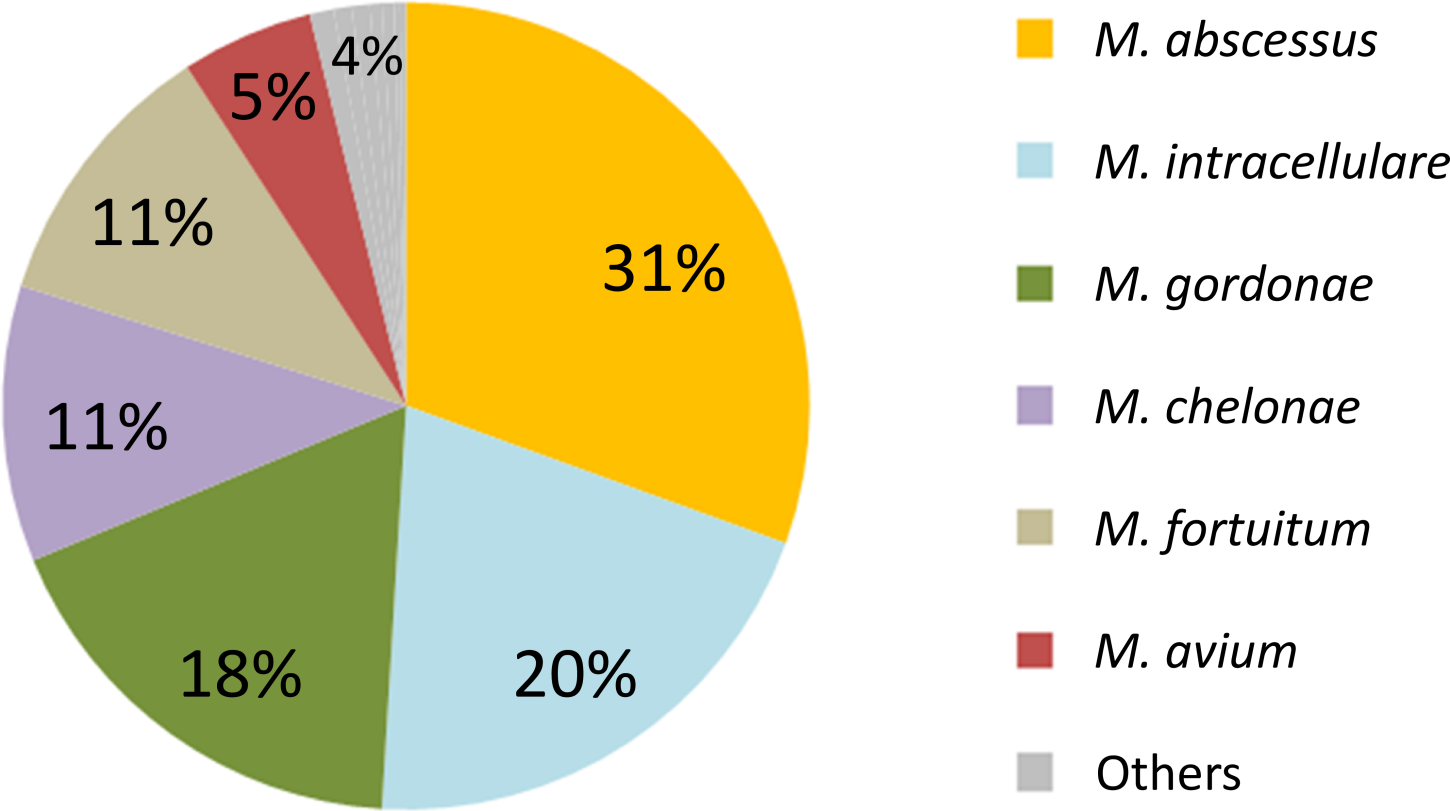
NTMs cultured from respiratory samples (N = 416). NTMs isolated from respiratory specimens were retrospectively analyzed. Others category includes: *Mycobacterium kansasii* (1.0%), *M*. *szulgai* (0.7%), *M*. *xenopi* (0.7%), *M*. *peregrium* (0.5%), *M*. *scrofulaceum* (0.5%), *M*. *simiae* (0.2%), and *M*. *terrae* (0.2%).

**Fig 2:**
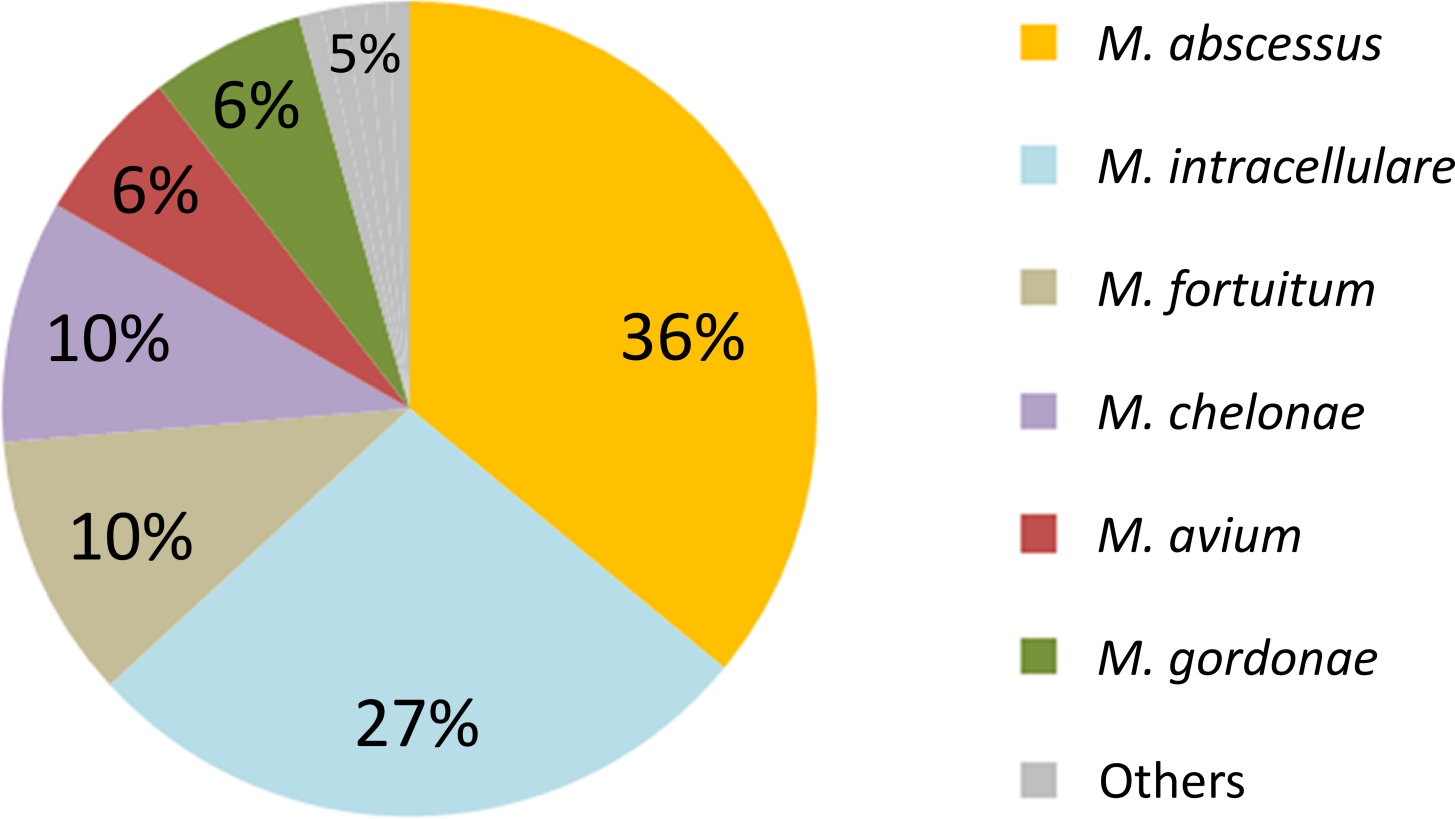
Epidemiology of NTM lung disease (N = 114). NTM lung disease cases, defined according to the American Thoracic Society criteria, were extracted from NTM detected cases shown in Fig 1. Others category includes: *Mycobacterium kansasii* (0.9%), *M*. *scrofulaceum* (0.9%), *M*. *szulgai* (0.9%), *M*. *terrae* (0.9%), and *M*. *xenopi* (0.9%).

Four hundred sixteen patients were found with positive NTM culture results from respiratory specimens. As shown in Fig 1, the most frequently detected NTM was MABC (n = 127; 30.5%), followed by *M*. *intracellulare* (n = 85; 20.4%). NTM lung disease was diagnosed, according to the ATS criteria, in 114/416 patients (27%). Fig 2 shows the etiology of NTM lung disease patients. Again, the most frequently detected pathogen was MABC (n = 41; 36.0%), followed by *M*. *intracellulare* (n = 31; 27.2%).

### Characteristics of NTM lung disease patients

Clinical background and symptoms were compared for patients diagnosed with either MABC or MAC (n = 41 and 38, respectively). No statistical differences in clinical background, except chronic obstructive pulmonary disease (COPD), tracheotomy, and cerebrovascular disease, were calculated between two groups (Table 1). COPD was more frequent in MABC patients compared with MAC patients (MABC 24% vs MAC 5%, p = 0.0265). Eight tracheotomized patients were included among NTM lung disease patients and all cases were infected with RGM; six of these cases were infected with MABC (MABC 15% vs MAC 0%, p = 0.0261), the remaining two were determined to be *M*. *chelonae*. These eight instances of tracheotomy were due to amyotrophic lateral sclerosis (n = 3), disturbed consciousness after operation (n = 1), old healed tuberculosis (n = 1), right lung hypoplasia (n = 1), post-operation of hypopharyngeal cancer (n = 1), symptomatic epilepsy after cerebral infarction (n = 1). Multivariate analysis using logistic regression determined patients with COPD were significantly more likely to develop MABC (Table 2). Observed symptoms were not found to be different between the two groups (Table 3).

**Table 1:** Patient background.

	MABC	MAC	
	[N = 41]	[N = 38]	*p* value
Age (median)	74	78.5	0.0567
Sex (male)	17 (41%)	12 (32%)	0.4840
Smoking history*	17 (41%)	14 (37%)	1.0000
Long-term care facility^✝^	6 (15%)	8 (21%)	0.5600
COPD	10 (24%)	2 (5%)	0.0265^§^
Bronchial asthma	7 (17%)	3 (8%)	0.3145
Old healed tuberculosis	7 (17%)	6 (16%)	1.0000
Bronchiectasis	6 (15%)	4 (11%)	0.7389
Interstitial lung disease	2 (5%)	3 (8%)	0.6675
Thoracic surgery	3 (7%)	3 (8%)	1.0000
Tracheostomy	6 (15%)	0	0.0261^§^
Ventilator	2 (5%)	1 (3%)	1.0000
Tracheal cannulation**	6 (15%)	1 (3%)	0.1101
Gastroesophageal disease	4 (10%)	3 (8%)	1.0000
Lung cancer	1 (2%)	0	1.0000
Other solid cancer	5 (12%)	4 (11%)	1.0000
Hematological cancer	1 (2%)	0	1.0000
Cardiovascular disease	16 (39%)	9 (24%)	0.1564
Chronic liver disease	4 (10%)	3 (8%)	1.0000
Chronic kidney disease	7 (17%)	6 (16%)	1.0000
Neuromuscular disease	3 (7%)	5 (13%)	0.4711
Cerebrovascular disease	2 (5%)	9 (24%)	0.0221^§^
Autoimmune disease	4 (10%)	8 (21%)	0.2148
Diabetes mellitus	6 (15%)	4 (11%)	0.7389
HTLV-1*	5 (12%)	1 (3%)	0.1409
Corticosteroid^#^	9 (22%)	8 (21%)	1.0000
Immunosuppressant	5 (12%)	2 (5%)	0.4338
Acid suppressant	23 (56%)	16 (42%)	0.2629

*Only 76 and 19 patients had data included for smoking history and HTLV-1, respectively.

^**✝**^Patients admitted from long-term care facilities.

**Tracheal cannulation: includes tracheotomized patients and orally intubated patients.

^**#**^Patients receiving daily corticosteroid at any dose.

^**§**^Considered significant.

Abbreviations: MABC; *Mycobacterium abscessus* complex, MAC; *Mycobacterium avium* complex, COPD; chronic obstructive pulmonary disease, HTLV-1; human T-lymphotropic virus 1

**Table 2:** Multivariate analysis to determine clinical charecteristics for MABC.

	Odds ratio	95% CI	*p* value
COPD	6.73	1.31–34.64	0.0225*
Tracheal cannulation**	6.45	0.69–60.44	0.1025
Bronchiectasis	1.56	0.36–6.71	0.5474
Gastroesophageal disease	1.23	0.22–6.96	0.8157
Old healed tuberculosis	0.91	0.24–3.44	0.8854

*Considered significant.

**Tracheal cannulation: includes tracheotomized patients and orally intubated patients.

Abbreviations: COPD; chronic obstructive pulmonary disease, CI; confidence interval

**Table 3:** Symptoms.

	MABC	MAC	
	[N = 41]	[N = 38]	*p* value
Fever (≧38°C)	9 (22%)	9 (24%)	1.0000
Cough	16 (39%)	18 (47%)	0.5009
Sputum	31 (76%)	27 (71%)	0.7995
Hemosputum	4 (10%)	5 (13%)	0.7312
Weight loss*	2 (5%)	0	0.4943

*Weight loss was defined as decrease of more than 5% of body weight in 6 months.

Abbreviations: MABC; *Mycobacterium abscessus* complex, MAC; *Mycobacterium avium* complex

### Chest CT findings

Chest CT was performed for 40 MABC and 36 MAC patients. Bronchiectasis, nodules, and consolidation were observed less frequently in MABC patients compared with MAC patients (Table 4).

**Table 4:** Chest CT findings.

	MABC	MAC	
	[N = 40]	[N = 36]	*p* value
Bronchiectasis	21 (53%)	29 (81%)	0.0150*
Nodules	14 (35%)	25 (69%)	0.0032*
Consolidation	17 (43%)	25 (69%)	0.0224*
Cavity	2 (5%)	3 (8%)	0.6631
Ground-glass opacity	9 (23%)	7 (19%)	0.7851
Linear scarring	30 (75%)	26 (72%)	0.8003

In total, 67 patients had more than one finding.

*Considered significant.

Abbreviations: MABC; *Mycobacterium abscessus* complex, MAC; *Mycobacterium avium* complex

## Discussion

MABC was revealed to be the most common causative pathogen for NTM lung disease in this cohort. However, since only two hospitals were involved in the current study, our data may not precisely reveal the etiology of NTM lung disease in Okinawa Prefecture due to sampling bias. However, our data indicates the detection rate of MABC among NTM lung disease patients in Okinawa was altogether different from previous Japanese reports. Morimoto et al., reported the epidemiology of NTM in Japan based on a laboratory-based analysis [13]. Although the data for Okinawa Prefecture was not isolated, MABC was detected more frequently in the Kyushu-Okinawa region than in other, more temperate, regions of Japan. Morimoto et al., also reports that MAC was the most common pathogen to cause NTM lung disease in the Kyushu-Okinawa region [10]. This result is markedly different from our data.

For convenience, Okinawa is frequently combined with Kyushu as the "Kyushu-Okinawa region," despite Okinawa being 700 kilometers away from the Kyushu district and falling within the subtropical zone. Due to the climate change and other potential factors, Okinawa experiences unique epidemiological events for many infectious diseases, which can be completely different from those experienced on mainland Japan [14–17]. Additionally, the population of Okinawa is only one tenth the size of Kyushu's (1.4 million vs 13 million). Therefore, we can assume that Okinawa’s distinctive epidemiology, observed in our study, was diminished in the aforementioned article. Moreover, the relatively high isolation rate of MABC in our study is similar to the epidemiology seen in other Asian countries [4, 5]. In Taiwan and South Korea, the isolation rate of MABC from NTM lung disease patients is higher [6, 18–21]. Frequently, differences in epidemiology can be explained by differences in climate conditions, environmental exposure, ethnic backgrounds, and laboratory practices (i.e., culture temperatures) [5]. The climate conditions of Okinawa and Taiwan are both classified as subtropical, however climate conditions in South Korea are decidedly different. Although further research is needed, common factors among South Korea, Taiwan, and Okinawa may be able to explain this characteristic epidemiology.

Our multivariate analysis showed a possible association between COPD and MABC infection. COPD was previously reported as a risk factor for NTM lung disease, however the distinct species, associated with COPD patients, was not specified [22]. As far as we know, risk factors related to the development of MABC lung disease remain unclear. However, past studies revealed that cystic fibrosis, gastroesophageal disease, and previous mycobacterial lung disease should be considered as potential risk factors related to the development of RGM induced lung disease [23, 24]. Since RGM induced lung disease is predominantly due to MABC [25], the risk factors mentioned above should also be considered risk factors for MABC lung disease. As with cystic fibrosis and previous mycobacterial lung disease, normal lung structure is destroyed and clearance of sputum is impaired in COPD, thus it is likely that COPD patients are susceptible to MABC infection, as also described by Chan et al. [26].

Since tracheostomy was not an appropriate variable for logistic regression in this study because of no tracheotomized patient in MAC group, tracheal cannulation was evaluated by multivariate analysis. Although the relationship between MABC and tracheal cannulation was not determined to be statistically significant, it is noteworthy that all tracheotomized patients with NTM lung disease had cultures positive for RGM (MABC: 6 cases, *M*. *chelonae*: 2 cases) in our study. Although information regarding the relationship between tracheotomy and MABC is limited, Do et al., reported that MABC was frequently detected from children with tracheostomy [27]. Their study retrospectively reviewed 5-years of data on MABC infection in a pediatric pulmonary center and 16 cases infected with MABC were identified. Of these, medical records were available for 15 patients and 73% of cases (11/15) were patients with tracheostomy. Lee et al., also showed MABC was frequently identified among ventilator-dependent patients [28]. Since RGM including MABC can cause skin and soft tissue colonization and infection, we speculate that MABC infection around tracheostomy may traverse the tracheal epithelium and eventually cause lung infection.

There is limited data concerning the clinical manifestations of MABC lung disease. Griffith et al., reported that cough was the most frequent symptom (70%) in RGM patients and hemoptysis and dyspnea were also common [25]. However, there are no RGM-specific respiratory symptoms, and our results also confirm this.

Our study demonstrated that bronchiectasis, nodules, and consolidation were observed more frequently in MAC patients than in MABC patients. According to Chung et al., no significant difference was observed in the presence of small nodules, tree-in-bud pattern, and bronchiectasis between two groups [29]. However, nodule, airspace consolidation, and thin-walled cavities were observed more frequently in MAC patients. Thus, results regarding bronchiectasis and cavitation were different from our study. Although the precise reason is unknown, one possible explanation is the difference of patients’ background. There are two major radiographic features of NTM lung disease; fibrocavitary form and nodular bronchiectatic form, with the former pattern often seen in older male patients with underlying lung disease such as COPD [7, 10]. Since COPD was more frequent in MABC patients compared with MAC patients in our study, bronchiectasis and nodules may be observed less frequently in MABC group. Another explanation for the differing radiographic findings may be the multiple subspecies of MABC. It has been reported that the etiological epidemiology of three subspecies of MABC (*M*. *abscessus*, *M*. *massiliense*, and *M*. *bolletii*) varies due to geographical region. Furthermore, differences in the genotypes of *M*. *abscessus* and *M*. *massiliense* were associated with varying radiological features and abnormal shadows in the lung [30, 31]. Since the distributional pattern of MABC genotypes is assumed different between Okinawa and South Korea, it is possible that the radiological features of MABC lung disease are also different.

Our study has some limitations. As a retrospective study conducted in two hospitals, our data is sparse and potentially biased. Data from several more hospitals in Okinawa should be collected to reveal a more accurate epidemiology of NTM lung disease in Okinawa. Additionally, we did not identify MABC to the subspecies level. According to DNA sequence and drug sensitivity, MABC is divided into 3 subspecies: *M*. *abscessus* (A-type), *M*. *massiliense* (M-type), and *M*. *bolletii* (B-type) [32–35]. Because some studies have shown differences among the biological characteristics, antibiotic sensitivity, and clinical features of the three subspecies of MABC [36, 37], differentiation of three subspecies of MABC may reveal more profound results.

Nevertheless, if treated as a pilot study, our results revealed considerable differences regarding the epidemiology of NTM lung disease in Okinawa, Japan and provided valuable insight regarding the differences between the clinical characteristics of MABC and MAC lung disease. Further studies are needed to present a more accurate understanding of the epidemiology, clinical features, and risk factors of MABC lung disease.

## Supporting information

S1 File Data for figures and tables. [Raw data for 2nd submission.xlsx].
(XLSX)
